# The Residence of the Infective Agent of Measles

**Published:** 1905-12-16

**Authors:** 


### Hospital Clinics.

### THE RESIDENCE OF THE INFECTIVE AGENT OF MEASLES.

There appeared recently in these columns a review of the results obtained from a detailed statistical investigation of the epidemic prevalence of measles in Aberdeen. As a not unfitting pendant to that report the following account of experimental measles may not come amiss, for it brings the history of the experimental production of measles practically up to date, although some months have elapsed since its publication.

The author, Ludvig Hektoen,[1] whose high authority in matters pathological is a guarantee of the scientific accuracy of the facts detailed, commences his contribution with an interesting review of the history of attempts to propagate the contagion of measles artificially. The earliest attempts at such a propagation appear to have been made at Edinburgh by Francis Home in 1758. This observer, arguing on the analogy afforded by the easy transmissibility of the contagion of variola, determined to attempt the inoculation of presumably susceptible subjects with blood derived from the superficial vessels of patients suffering from measles. The contemporary view of the localisation of the virus seems to be indicated by the term applied to the blood. Hare speaks of it as the "magazine of all epidemic diseases," and urges that the blood to be used for inoculation should be taken from areas of the skin where the eruption of the disease is most in evidence. The inoculation was carried out precisely as for vaccination. Home made fifteen attempts and records his belief that he succeeded in producing a mitigated form of measles. For a long while Home's results were accepted as establishing the inoculability of measles, but in 1817 two Dutch observers, having carefully adhered to the technique prescribed by Home, achieved nothing but negative results. Not only so, but they even throw doubt upon Home's claim to have performed the experiments. It appears, however, that the experiments almost certainly were performed, but Hektoen, after a critical review of the facts recorded by Home, is compelled to the conclusion that in all probability not one of Home's experimental patients really contracted measles. "In support of this view," says he, "I may point out that in no single case is the period between the inoculation and the appearance of the rash given as more than ten days, but generally as less, and even so short as seven days, whereas we now know definitely that the period between exposure and eruption in measles is from thirteen to fourteen days." Moreover, one of Home's successful cases actually "took measles again" a few weeks later, which adds corroboration to suspicions of his accuracy.

In 1822 Speranza, of Mantua, made a series of what purported to be successful inoculations. But in them, again, the incubation period was very short, varying in length from five to six days. So brief an interval of incubation transgresses our knowledge of the disease sufficiently to prompt Hektoen to refuse credence to the series.

In 1842 Katona in Hungary made 1,122 inoculations during an epidemic, and claimed to have achieved as much as 93 per cent. of successes. But here again Hektoen is sceptical of the true nature of the transmitted disorder. The details are scanty: the subjects of the experiments were living in the centre of an epidemic focus of the disease, and we are not told of any effort to safeguard them from natural infection in the ordinary way; lastly, the average incubation period was no more than seven days.

The most credible series of experiments in this direction seem to be those carried out by F. Mayr. During an epidemic of measles occurring in Vienna in the years 1845 to 1851 Mayr vaccinated six patients successfully, using for his inoculation the material obtained by scratching the centre of a rubeolous spot: this means that he did not, as he claimed to do, employ nothing but the blood of the affected patient, for his vaccine no doubt contained both epidermal scales and tissue juices. In three other cases he introduced on to the nasal mucous membranes of the subjects of his experiments fresh mucus derived from the noses of measly patients. In two of these three cases a typical measles rash appeared on the fourteenth day after infection.

From this date interest in the artificial transmission of measles appears to have languished. Hektoen himself set out to determine by experiment if possible whether or not during an attack of measles the blood of the patient contained the infecting agent at the height of the attack. His experiments were two in number, and to the following effect.

The first source of "vaccine" was a boy of nine years, who, in the later stages of desquamation after an uncomplicated attack of scarlet fever, developed mild but typical measles. He had been free from the fever associated with the earlier disease for two weeks when he was attacked by headache, cough, coryza, running at the eyes, and mild febrile symptoms. Three days later there appeared a papular eruption, followed on the next day by a typical rubeolous rash which was succeeded by a branny desquamation. On the fourth day of this attack of measles four cubic centimetres of blood were withdrawn from a vein at his right elbow under the strictest aseptic precaution. With this blood two culture-tubes of peptone-broth were inoculated, and incubated at body-temperature for twenty-four hours. But both cultures remained sterile as far as ordinary evidence of growth was concerned. Sub-cultures also remained sterile though implanted upon a variety of media. After twenty-four hours of incubation at body-heat four cubic centimetres of a mixture of such broth and blood were injected beneath the skin of the chest of a healthy medical student who readily offered himself for the experiment. This man was twenty-four years old, and was in the later stages of peeling after an uncomplicated attack of scarlet fever.

He had been for twenty-six days in an institution free of measles, and had himself never previously had anything at all like measles except the scarlet fever which had brought him there. On the thirteenth day after his inoculation his temperature rose to 101° F., the next day it reached 103° F., and on the third day, after having taken a warm bath, he manifested a red papular blotchy eruption which, appearing first on his forehead, spread rapidly to his face, neck and chest. His temperature remained slightly raised for a couple of days, and fell as the rash began to fade. There followed a branny desquamation such as is commonly associated with measles.

The source of the vaccine in the second experiment was a girl of twenty-one years who underwent a typical bout of measles. While the rash was still coming out some blood was withdrawn from a vein at her elbow in a fashion precisely simliar to, and with precautions identical with, those adopted in the previous case. This blood was, as before, incubated in sterile broth for twenty-four hours, and then injected into a subject who had never before had measles. This man, on the eleventh day following the injection, showed a slight rise of temperature, on the twelfth day a mild conjunctivitis, on the thirteenth day a cough, and on the fourteenth day a typically morbilliform rash.

The conclusion drawn by Hektoen from these two experiments is that the virus of measles is present in the blood of patients who present the typical appearances of measles some time at least during the first thirty hours of the eruption. Furthermore, that the virus retains its virulence for at least twenty-four hours when mixed with sterile broth and maintained at a temperature of 37° C. This being so, the experiments go to prove that it is not difficult to obtain the virus of measles uncontaminated with other infections and thus in a form available for study by many different methods.

[1] Jour. of Infec. Dis., Chicago, March 1905.

